# Detection of prions in skin punch biopsies of Creutzfeldt–Jakob disease patients

**DOI:** 10.1002/acn3.51000

**Published:** 2020-03-06

**Authors:** Angela Mammana, Simone Baiardi, Marcello Rossi, Alessia Franceschini, Vincenzo Donadio, Sabina Capellari, Byron Caughey, Piero Parchi

**Affiliations:** ^1^ IRCCS Istituto delle Scienze Neurologiche di Bologna Bologna Italy; ^2^ Department of Biomedical and Neuromotor Sciences University of Bologna Bologna Italy; ^3^ LPVD Rocky, Mountain Laboratories NIAID NIH Hamilton Montana; ^4^ Department of Experimental, Diagnostic and Specialty Medicine University of Bologna Bologna Italy

## Abstract

Prion real‐time quaking‐induced conversion (RT‐QuIC) is an ultrasensitive assay detecting pathological aggregates of misfolded prion protein in biospecimens. We studied 71 punch biopsy skin samples of 35 patients with Creutzfeldt–Jakob disease (CJD), including five assessed in vitam. The results confirmed the high value of skin prion RT‐QuIC for CJD diagnosis (89% sensitivity and 100% specificity) and support its use in clinical practice. Preliminary data based on a limited number of cases suggest that prion‐seeding activity in the skin varies according to the prion strain, being higher in sporadic CJD subtypes linked to the V2 strain (VV2 and MV2K) than in typical CJDMM1.

## Introduction

The recent introduction of in vitro assays based on the ultrasensitive, amplified measurement of pathological protein aggregates in biospecimens provided novel accurate diagnostic methods for protein‐folding diseases such as prion diseases and synucleinopathies.^1^ Among them, the prion real‐time quaking‐induced conversion (RT‐QuIC) assay is emerging as the most accurate test for the in vivo diagnosis of Creutzfeldt–Jakob disease (CJD),^2^, ^3^, ^4^ the most common human prion disease. CJD comprises six major subtypes with highly heterogeneous clinico‐pathological phenotypes, which are largely determined and classified by the genotype at the polymorphic codon 129 (encoding methionine, M or valine, V) in *PRNP*, and by the type (type 1 or type 2) of abnormal prion protein (PrP^Sc^) accumulating in the brain (e.g., MM1, VV1, MM2, VV2, MV2).^5^

The RT‐QuIC test is currently mainly applied to cerebrospinal fluid (CSF) but attempts to adapt the assay to tissue sources that might be easier to collect or represent an alternative procedure in patients with contraindications to lumbar puncture are currently being pursued. Recently, Orrù et al. detected prion‐seeding activity in post‐mortem skin samples of CJD patients with an overall sensitivity and specificity of 100%.^6^ In the present study, we aimed to validate the diagnostic accuracy of skin RT‐QuIC in another patient series including different disease subtypes and a few samples taken in vitam. Furthermore, to improve the clinical applicability of the assay, we focused on small punch biopsy samples and applied a fast tissue homogenization protocol lacking time‐consuming purification or concentration steps.

## Patients and Methods

### Inclusion criteria and case classification

We studied a consecutive series of 57 patients referred for diagnosis because of the clinical suspicion of CJD and an additional control group of 15 patients with various neurological disorders who were investigated for a possible small fiber neuropathy (diabetic neuropathy [*n* = 3], idiopathic small fiber neuropathy [*n* = 4], pure autonomic failure [*n* = 1], Parkinson’s disease [*n* = 2], Alzheimer’s disease [*n* = 2], and Dementia with Lewy bodies [*n* = 3]).

Prion disease (*n* = 35) was diagnosed according to the current European criteria for CJD (https://www.cjd.ed.ac.uk/sites/default/files/criteria_0.pdf). The non‐CJD group included all clinically suspected cases resulting prion‐negative at post‐mortem examination (*n* = 22) as well as the “clinical” cases (*n* = 15) investigated in vivo. Molecular analysis of the *PRNP* gene, PrP^Sc^ typing, and subtype classification were performed in all prion‐positive subjects as described (Table 1).^7^

**Table 1:** Demographic features and results of standard diagnostic investigations in the tested patient cohort.

**Final diagnosis**	***n***	**Mean, SD**		**CSF**			**Brain MRI**
		Age at onset, y	Disease duration, mo	14‐3‐3^a^ Pos./tested (%)	t‐tau^b^ Pos./tested (%)	RT‐QuIC^c^ Pos./tested (%)	Pos.^d^/tested (%)
**Definite sCJD**	**29**	**70.8 ± 11**	**8.2 ± 9.3**	**19/23 (82.6)**	**23/24 (95.8)**	**18/21 (85.7)**	**20/25 (80.0)**
MM1	21	70.7** ± **11	6.2** ± **9.2	14/17 (82.4)	18/18 (100)	14/16 (87.5)	13/18 (72.2)
VV2	3	70.0** ± **14	8.9** ± **4.8	1/1 (100)	1/1 (100)	1/1 (100)	3/3 (100)
MV2K	2	68 ‐ 78	5.1 ‐ 15.4	2/2 (100)	2/2 (100)	1/2 (50.0)	2/2 (100)
MM2C	3	70.7** ± **8	20.5** ± **8.9	2/3 (66.7)	2/3 (66.7)	2/2 (100)	2/2 (100)
**Probable sCJD**	**3**	**64.0 ± 4**	**7.8 ± 6**	**2/3 (66.7)**	**1/1 (100)**	**3/3 (100)**	**3/3 (100)**
MM	1	60	NA^e^	1/1 (100)	1/1 (100)	1/1 (100)	1/1 (100)
MV	1	65	12.0	0/1 (0)	1/1 (100)	1/1 (100)	1/1 (100)
VV	1	67	3.5	1/1 (100)	1/1 (100)	1/1 (100)	1/1 (100)
**Genetic CJD**	**3**	**70.0 ± 9**	**5.7 ± 6.9**	**3/3 (100)**	**3/3 (100)**	**3/3 (100%)**	**2/2 (100)**
E200K‐129M	2	63 ‐ 69	2.4 ‐13.7	2/2 (100)	2/2 (100)	2/2 (100)	2/2 (100)
V210I‐129M	1	80	1.1	1/1 (100)	1/1 (100)	1/1 (100)	NA
**Non‐CJD**	**37**	**67.8 ± 11**	**25.2 ± 50** f	**7/12 (58.3)**	**5/14 (35.7)**	**0/12 (0)**	**5/21 (23.8)**

The bold character highlights the main table headings and the main patient groups (the other rows are further distinctions/sub‐classification within each patient group).

CSF, cerebrospinal fluid; MRI, magnetic resonance imaging; sCJD, sporadic Creutzfeldt–Jakob disease; NA, not available.

a Evaluated semi‐quantitatively by Western blotting as described.^13^

b Measured quantitatively by ELISA as described.^13^

c Performed as described.^13^

d According to the current European diagnostic criteria for CJD diagnosis.^13^

e The patient is still alive.

f Calculated in the patient group referred for suspected CJD.

### Skin biopsy and sample preparation

Skin tissues were collected using a 3‐mm punch in the cervical C7 paravertebral area (proximal site) and/or in the lateral surface of the thigh (~20 cm above the patella) (distal site).^8^ Ex vivo samples were taken at autopsy, before opening the skull to avoid cross‐contamination with brain tissue. Skin samples were washed three times in cold 1 × PBS, homogenized at 1% (w/v) with gentleMACS Octo Dissociator (Miltenyi BioTec) in cold QuIC buffer (150 mmol/L NaCl, 4 mmol/L EDTA, 1 × PBS, Complete Protease Inhibitor Mixture) and stored at −80°C.^9^ 10% (w/v) brain homogenates in cold QuIC buffer with 1% Triton X‐100 of a sCJDVV2 and an AD subject were used as additional positive and negative controls.

### PrP^Sc^ detection by RT‐QuIC

Samples were analyzed using two different substrates, namely full‐length Syrian hamster (Ha23‐231) and Bank vole (Bv23‐230) rec‐PrP. The latter was produced in house, while Ha23‐231 was obtained by Dr. A. Green, University of Edinburgh. Both substrates were purified according to a previously published protocol.^10^, ^11^

After thawing, samples were centrifuged at 1000*g* for 10 min at 4°C and supernatants were serially diluted in N2 buffer (0.1% or 0.05% SDS, respectively, for Ha23‐231 and Bv23‐230 in 1 × PBS supplemented with N2 media [Gibco]) up to a final concentration of 10^−5^ for brain and 10^−3^ for skin samples. Diluted samples were added to the reaction mix, loaded in quadruplicate in a black 96‐well plate with a clear bottom (Nunc) and incubated in a FLUOstar OMEGA reader (BMG, Germany) over a period of 60 h as previously described.^12^ The fluorescence intensity of ThT‐PrP^Sc^ aggregates, expressed as relative fluorescence units (RFU), was taken every 45 min with a gain of 2000 for Ha23‐231, and of 1600 for Bv23‐230. Sample was considered prion‐positive if at least two out four sample replicates gave a fluorescence signal higher than the threshold cut‐off value. This threshold indicated the mean of RFU values of all negative samples at 60 h plus 5 or 20 standard deviations for Ha23‐231 and Bv23‐230, respectively. The following variables were used to quantitate the RT‐QuIC output: the time taken by the signal to reach the threshold (lag phase), the maximum fluorescence read (ThT max), and the area under the curve (AUC) representing the progression of the signal during the 60 h.

### Statistical analyses

RT‐QuIC relative fluorescence responses were analyzed and plotted using the Sigma Plot software (Systat Software Inc., Chicago, IL). The Mann–Whitney test was used to reveal differences between two groups; a *P* value <0.05 was considered statistically significant. Unless stated otherwise, data are expressed as mean ± standard error of the mean (SEM).

## Results

Demographic data and results of diagnostic investigations for the patient cohort are summarized in Table 1 **.**

### Accuracy of prion detection in skin punch biopsies across the spectrum of human prions

**Figure 1:**
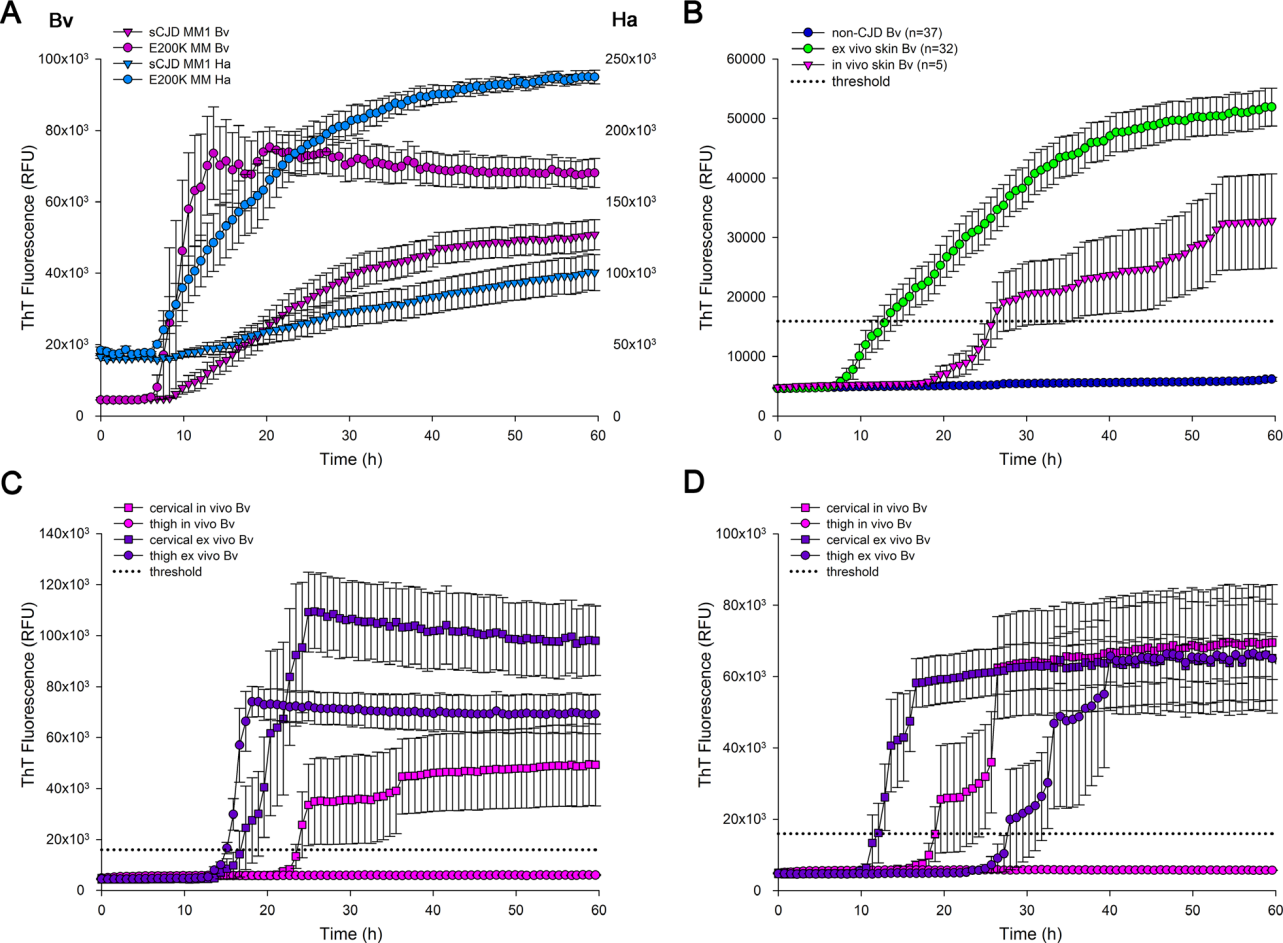
PrP^Sc^ detection by real‐time quaking‐induced conversion assay (RT‐QuIC). Traces represent the mean of the ThT fluorescence signal expressed in relative fluorescence unit (RFU) ± SEM. In particular, the “Bv” and “Ha” axes show the scale of RFU values obtained using the Bv23‐230 and Ha23‐231 rPrP, respectively. (A) The gCJD‐E200K skin samples showed a significant higher ThT response than that from sCJDMM1 cases using both bank vole (Bv, ThT max: gCJD‐E200K 82963 ± 9927 vs. sCJDMM1 53570 ± 4281, *P* < 0.05; AUC: gCJD‐E200K 3602549 ± 338362 vs. sCJDMM1 1940344 ± 199141 *P* < 0.05) and hamster (Ha, ThT max: gCJD‐E200K 238146 ± 4611 vs. sCJDMM1 98790 ± 11397, *P* = 0.005; AUC: gCJD‐E200K 10525425 ± 544543 vs. sCJDMM1 4203555 ± 485585, *P* = 0.005) prion protein as substrate for the RT‐QuIC reaction. (B) Ex vivo skin samples (*n* = 32) showed a significant overall higher fluorescence response and AUC and a slightly earlier lag phase than in vivo samples (*n* = 5) (ThT max: ex vivo 54861 ± 3440 vs. in vivo 33789 ± 7751, *P* < 0.05. AUC: ex vivo 1964412 ± 151226 vs. in vivo 1072265 ± 223689 *P* < 0.05). (C) In a sCJDMM1 case, both cervical and thigh ex vivo skin samples showed an earlier lag phase (17.4 h and 15.1 h, respectively) and a higher fluorescence response (ThT max: 113441 ± 15971 and 75662 ± 6342; AUC: 4158519 ± 586601 and 3177825 ± 308747) than the only in vivo skin sample tested positive (cervical site, lag phase 24.1 h; ThT max: 49810 ± 16144; AUC: 1710721 ± 560564). (D) In a sCJDMV2K case, both the ex vivo skin samples and the in vivo skin specimen from the cervical site tested positive with similar maximum ThT response and AUC (ThT max: cervical ex vivo 66565 ± 6283, thigh ex vivo 67010 ± 15748, cervical in vivo 70589 ± 4681; AUC: cervical ex vivo 2983687 ± 253835, thigh ex vivo 1936585 ± 457878, cervical in vivo 2580297 ± 282050), but different lag phases (12.0 h, 28.0 h and 19.6 h, respectively).

To this aim, we initially compared the performance of the two recombinant substrates. Although both protocols demonstrated a 100% specificity in distinguishing CJD from non‐CJD cases, the Bv23‐230 substrate showed an overall better sensitivity, due to higher accuracy in detecting PrP^Sc^ seeds in the most common sporadic (s)CJDMM1 subtype (85.7% vs. 52.4% for Ha23‐231) (Table 2). Indeed, the sensitivity of the test for sCJDVV2 and MV2K samples was 100% using both protocols. This result suggests that in these subtypes, the skin prionseeding activity is significantly higher than in sCJDMM1. Interestingly, as previously demonstrated for CSF,^4^, ^13^ genetic (g)CJD E200K samples showed a slightly earlier and a statistically significant higher signal response than those of sCJDMM1, using both substrates (Fig. 1A).

**Table 2:** Sensitivity and specificity of the skin RT‐QuIC assay across the CJD spectrum using Ha23‐231 or Bv23‐230 rec‐PrP as substrate.

	**Ha23‐231**		**Bv23‐230**	
	**Cervical**	**Thigh**	**Cervical**	**Thigh**
**Ex vivo**	**Positive/tested (%)**			
sCJD MM1^a^	8/20 (40.0)	8/19 (42.1)	17/20 (85.0)	13/19 (68.4)
sCJD VV2	3/3 (100)	2/3 (66.7)	3/3 (100)	3/3 (100)
sCJD MV2K	2/2 (100)	2/2 (100)	2/2 (100)	2/2 (100)
sCJD MM2C	2/3 (66.7)	2/3 (66.7)	2/3 (66.7)	2/3 (66.7)
gCJDE200K‐129M	2/2 (100)	2/2 (100)	2/2 (100)	2/2 (100)
gCJDV210I‐129M	1/1 (100)	0/1 (0)	1/1 (100)	1/1 (100)
non‐CJD^b^	0/19 (0)	0/18 (0)	0/19 (0)	0/18 (0)
**In vivo**				
sCJD MM^c^	1/2 (50.0)	0/2 (0)	2/2 (100)	0/2 (0)
sCJD MV^d^	2/2 (100)	1/2 (50.0)	2/2 (100)	1/2 (50.0)
sCJD VV	0/1 (0)	1/1 (100)	0/1 (0)	1/1 (100)
non‐CJD^e^	0/10 (0)	0/12 (0)	0/10 (0)	0/12 (0)
**Sensitivity**	**24/35 (68.6)**		**31/35 (88.6)**	
**Specificity**	**0/37 (100)**		**0/37 (100)**	

The bold character highlights the main table headings and the main conclusive results (sensitivity and specificity of the assay).

Ha23‐231, full‐length hamster PrP residues 23‐231; Bv23‐230, full‐length bank vole PrP residues 23‐230; sCJD, sporadic Creutzfeldt–Jakob disease; gCJD, genetic Creutzfeldt–Jakob disease.

a The cervical sample was not available in one case, the thigh sample in two.

b The cervical sample was not available in three cases, the thigh sample in four.

c Includes also the definite sCJDMM1 case examined both in vivo and ex vivo.

d Includes also the definite sCJDMV2K patient examined both in vivo and ex vivo.

e The cervical sample was not available in five cases, the thigh sample in three.

### RT‐QuIC detection of prion in skin punch biopsies taken in vivo

With the Bv substrate, all five sCJD patients examined in vivo gave a positive response in at least one of the two sites, whereas all non‐CJD samples yielded a negative response, confirming the full specificity of the assay (Table 2). Four out of five sCJD cervical samples showed a positive fluorescence response, while one patient showed a positivity limited to the thigh. Among the four cases that were positive in the cervical site, only one also gave a positive reaction in the thigh. Interestingly, the direct comparison between positive ex vivo and in vivo samples showed a significantly higher maximum ThT fluorescence and AUC and a slightly shorter lag phase in the former group (Fig. 1B).

### The timing of sample collection affects skin RT‐QuIC reactivity

In three probable CJD cases, skin samples were collected only in vivo at 3 (VV), 11 (MV), and 20 (MM) months from clinical onset, while two subjects underwent both in vivo and ex vivo punch biopsies. In a sCJDMM1 patient, the samples taken in vivo 1 month from clinical onset gave a positive response, albeit not full, in the cervical area (3/4 wells), and a negative one in the thigh. The ex vivo samples, taken 34 months later, gave a full positivity in both areas, with a significantly higher fluorescence response and a shorter lag phase (Fig. 1C).

In a sCJDMV2K patient, the samples collected 11.5 months from onset gave a partial positive response limited to the cervical area (3/4 wells). The ex vivo samples collected 1 month later, yielded an entirely positive result in both regions with a shorter lag phase in the cervical area (Fig. 1D).

## Discussion

The present results confirm the high diagnostic accuracy of skin prion RT‐QuIC and support its use either in combination with CSF analysis or as an alternative to the CSF assay in patients with contraindications to lumbar puncture. Most significantly, our data show that prion‐seeding activity can be detected in the majority of patients using a limited amount of tissue and a minimally invasive and innocuous procedure. In particular, from the preliminary data obtained in vivo, the cervical area appears the preferable skin site because of its more consistent positive response.

With the significant limitation of the low number of tested CJD cases apart from the MM1 group, our results also provide preliminary data about the issue of prion replication outside the CNS and its relationship with prion strains. Interestingly, the lower performance of the Ha PrP substrate with samples from sCJDMM1 subjects is consistent with previous findings from our group indicating that the M1 CJD strain is associated with a lower amount of PrP^Sc^‐seeding activity in peripheral tissues in comparison to the V2 strain, linked to the VV2 and MV2K subtypes.^9^ Thus, we speculate that the amount of PrP^Sc^ deposition in the skin of sCJDVV2 and MV2K would be well above the limit of detection of the assays and, consequently, sufficient to always seed the reaction with both protocols despite the higher reactivity of the Bv23‐230 substrate, while this would not be the case for the sCJDMM1 samples. The more significant and possibly earlier reactivity in cervical samples, in particular in those patients analyzed both in vivo and ex vivo, seem to support the view of a centrifugal spread from the CNS to the periphery rather than a centripetal course. Although very preliminary, our data also suggest that prion‐seeding activity in the skin increases over time with disease progression. In conclusion, skin punch biopsies may represent a valid alternative to CSF to demonstrate a positive prion‐seeding activity in suspected prion diseases. The ultrasensitive detection of prion‐seeding activity in skin samples at the clinical disease stage does not have any obvious implication regarding infectivity and transmission risk, although the issue may deserve further investigations.

## Conflict of Interest

BC is an inventor on patents related to RT‐QuIC assays. The other authors have nothing to report.

## Authors’ Contributions

A.M, S.B., and P.P. contributed to conception and design of the study. All authors contributed to acquisition and/or data analysis. A.M., S.B., A.F., and P.P. contributed to drafting the text and preparing the figure. PP supervised the study.

## Data Availability

The datasets generated during and/or analyzed during the current study are available from the corresponding author on reasonable request.
